# Novel frameshift mutations of ANKUB1, GLI3, and TAS2R3 associated with polysyndactyly in a Chinese family

**DOI:** 10.1002/mgg3.1223

**Published:** 2020-04-06

**Authors:** Lishan Zhang, Xiaobin Chen, Lanwei Xu, Shibing Guan, Dehua Wang, Yanliang Lin, Zengtao Wang

**Affiliations:** ^1^ Department of Hand and Foot Surgery Shandong provincial Hospital Affiliated to Shandong University Jinan China; ^2^ Center Laboratory Shandong provincial Hospital Affiliated to Shandong University Jinan China

**Keywords:** ANKUB1, GLI3, mutation, polysyndactyly, TAS2R3

## Abstract

**Background:**

Polysyndactyly (PSD) is an autosomal dominant genetic limb malformation caused by mutations.

**Methods:**

Whole exome sequencing and Sanger sequencing were used to determine the mutations in PSD patients. Luciferase reporter assay was performed to determine the effect of GLI3 mutation on its transcriptional activity.

**Results:**

In this study, we investigated the gene mutations of three affected individuals across three generations. The frameshift mutations of GLI3 (NM_000168:c.4659del, NP_000159.3: p.Ser1553del), ANKUB1 (NM_001144960:c.1385del, NP_001138432.1: p.Pro462del), and TAS2R3 (NM_016943:c.128_131del, NP_058639.1: p.Leu43del) were identified in the three affected individuals, but not in three unaffected members by whole exome sequencing and sanger sequencing. Luciferase reporter assay demonstrated that *GLI3* mutation reduced the transcriptional activity of GLI3. The results from SMART analysis showed that the frameshift mutation of *TAS2R3* altered most protein sequence, which probably destroyed protein function. Although the frameshift mutation of *ANKUB1* did not locate in ankyrin repeat domain and ubiquitin domain, it might influence the interaction between ANKUB1 and other proteins, and further affected the ubiquitinylation.

**Conclusion:**

These results indicated that the frameshift mutations of *GLI3, ANKUB1,* and *TAS2R3* might alter the functions of these proteins, and accelerated PSD progression.

## Graphical abstract

In this study, we investigated the gene mutations of three affected individuals across three generations. The frameshift mutations of GLI3, ANKUB1, and TAS2R3 were identified in the three affected individuals, but not in three unaffected members by whole exome sequencing and Sanger sequencing.

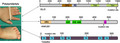

## 1 INTRODUCTION

Polysyndactyly (PSD) is an autosomal dominant genetic limb malformations characterized by incomplete penetrance and phenotypic variability, and can occur independently or as part of a syndrome (Malik et al., 2006). PSD is significantly clinical heterogeneity, involving unilateral or bilateral limb, and presents symmetrical or asymmetric malformation (Brison, Debeer, & Tylzanowski, 2014). PSD displays complex genetic heterogeneity, implicating multiple genes in various pedigrees in different populations. Several gene mutations, including *HOXD13* (OMIM142989), *GLI3* (OMIM165240), *GJA1* (OMIM121014), *FBLN1* (OMIM135820), *Lmbr1* (OMIM605522), and *SHH* (OMIM600725), were verified as direct causes of PSD (Brison et al., 2014; Debeer et al., 2002; Hui & Angers, 2011; Richardson, Donnai, Meire, & Dixon, 2004; Robertson, Tickle, & Darling, 1997; Wang et al., 2007). The GLI3 protein is a zinc finger transcription factor expressed early in development of vertebrates (Al‐Qattan, 2012). *GLI3* mutations lead to a variety of clinical phenotypes, such as Greig cephalopolysyndactyly syndrome (GCPS, OMIM175700), Pallister–Hall syndrome (PHS, OMIM146510), Acrocallosal syndrome (ACLS, OMIM200990), preaxial polydactyly type IV (PPD4, OMIM174700), and postaxial polydactyly type A (PAPA1, OMIM174200) (Shin, Kogerman, Lindstrom, Toftgard, & Biesecker, 1999). Remarkably, mutations in several sites of GLI3 have been identified in different pedigrees with PSD (Al‐Qattan, Shamseldin, Salih, & Alkuraya, 2017). In this study, we evidenced a novel heterozygous frameshift mutation of *GLI3* associated with PSD in a Chinese family. In addition, novel heterozygous frameshift mutations of *ANKUB1* and *TAS2R3* (OMIM604868) were identified in this pedigree.

## 2 MATERIALS AND METHODS

### 2.1 Patients and ethical compliance

A three‐generation Chinese family that contains three patients with limb malformation and three healthy individuals was investigated. Venous blood samples were obtained from affected and unaffected family members. Genomic DNA was isolated and quantified using Qubit® DNA Assay Kit in Qubit® 2.0 Flurometer (Life Technologies). This study was approved by the Ethics Committee of Shandong provincial Hospital affiliated to Shandong University, and informed consent was obtained for experimentation with human subjects.

### 2.2 Library preparation and sequencing

Genomic DNA was sheared into 150–200 bp fragments by hydrodynamic shearing system (Covaris). Remaining overhangs were converted into blunt ends using exonuclease/polymerase activities. After adenylation, DNA fragments were ligated with adapter oligonucleotides on both ends, and were selectively enriched via a PCR reaction, followed by hybridization with biotin‐labeled probe. Magnetic beads with streptomycin were used to capture the exons of genes. DNA libraries were enriched in a PCR reaction and sequenced on Illumina Hiseq platform.

### 2.3 Variant analysis

Valid sequencing data was mapped to the human reference genome sequence from UCSC database by Burrows–Wheeler Aligner (BWA) software (Li & Durbin, 2009–1). Single‐nucleotide polymorphisms (SNPs) and insertions–deletions (InDels) were identified by Samtools and GATK software (Li et al., 2009–2). 1,000 Genomes databases and dbSNP databases were used to characterize the detected variants. ANNOVAR (Wang, Li, & Hakonarson, 2010) was performed to annotate SNPs and InDels. Gene transcript annotation databases, including Consensus CDS, RefSeq, Ensembl, and UCSC, were used to determine amino acid alternation. Sorting Intolerant from Tolerant (SIFT) and Polymorphism Phenotyping version 2 (PolyPhen‐2) were performed to assess the functional relevance of the detected variants.

### 2.4 Sanger sequencing

The selected mutations were verified by PCR combined with Sanger sequencing. The primers for amplifying DNA fragments containing mutation sites are listed in Table 1.

**Table 1:** Sequences of the primers used for gene mutations amplification and sequencing

Gene name	Forward primer	Reverse primer
OR8U1	5'‐GCCTCTCCTATTGCCACTCC‐3'	5'‐GAGAGCCACACGTCGAGAAA‐3'
SP110	5'‐CAGCATTCGTGGGTCTCCAT‐3'	5'‐CCGTGCTTCCTGTCTTTCCT‐3'
ANKUB1	5'‐AAGCCCTCAAATTTCATCCAC‐3'	5'‐CCTTGCCACAGCTAAGCAGTAG‐3'
TAS2R3	5'‐CAGGGCTGCCTAATTGCTGA‐3'	5'‐TTCCTGGTGGCCTCAATTCC‐3'
GLI3	5'‐TACTTTCCCCAGGTGCTAATCA‐3'	5'‐TGAAACACATCTCAGTTAGGTG‐3'

### 2.5 Construction of GLI3 truncation mutation

Full‐length human GLI3 cDNA was obtained by PCR amplification using the specific primers (CTAGCGGCCGCGCCACCATGGAGGCCCAGTCC; GCTCTAGA TCCTATTGATTTCCGTTGG). According to the results from variant analysis, GLI3 truncation mutation was generated by PCR amplification using the specific primers (CTAGCGGCCGCGCCACCATGGAGGCCCAGTCC; GCTCTAGA TCATGTCCCCGATAGCC). The fragments were digested by Not I and Xba I, and were cloned into flag‐tagged pcDNA3 vector (Addgene) to construct pcDNA3.1‐GLI3‐WT (wild type) and pcDNA3.1‐GLI3‐MT (mutation type).

### 2.6 Luciferase reporter assay

The 1256‐bp fragment of PTCH1 5’UTR (potistion −2974 to −1718) was amplified using forward (5'‐AGCGGTGTTGTCTGGTAG‐3') and reverse (5'‐AATCGCCTTTCTTGAGTG‐3') primers. The PCR product was cloned into the pGL3 basic vector (Promega, Madison, USA) to construct pGL3‐PTCH1. 293T cells were co‐transfected with 0.5‐μg pGL3‐PTCH1 and indicated concentration of pcDNA3.1‐GLI3‐WT or pcDNA3.1‐GLI3‐MT using Lipofectamine 3,000 (Roche). The pRL‐SV40 plasmid (Promega) was used for a normalizing control. After 48 hr of incubation, luciferase activities were determined using the Dual‐Luciferase Assay (Promega) according to the manufacturer's instructions.

### 2.7 Western blot analysis

The transfected cells were lysed using RIPA lysis buffer (Thermo) to obtain total protein. The protein concentration was measured using the bicinchoninic acid method. Equal amounts of proteins were loaded on 8% SDS‐PAGE and transferred to PVDF membranes (EMD Millipore). The membranes were blocked for 1 hr with 5% nonfat milk, and were then incubated with the indicated primary and secondary antibodies. The protein signals were visualized using the enhanced chemiluminescence method and quantified using Scion Image 4.03 software.

### 2.8 Statistical analysis

Statistical analysis was carried out using GraphPad Prism 5.0. All data are presented as mean ± *SD* from at least three independent experiments. The Student's *t* test was used to assess the difference between two groups. One‐way ANOVA was used to assess the difference among multiple groups. *p* < .05 was considered as statistical significance.

## 3 RESULTS

### 3.1 Clinical features

**Figure 1:**
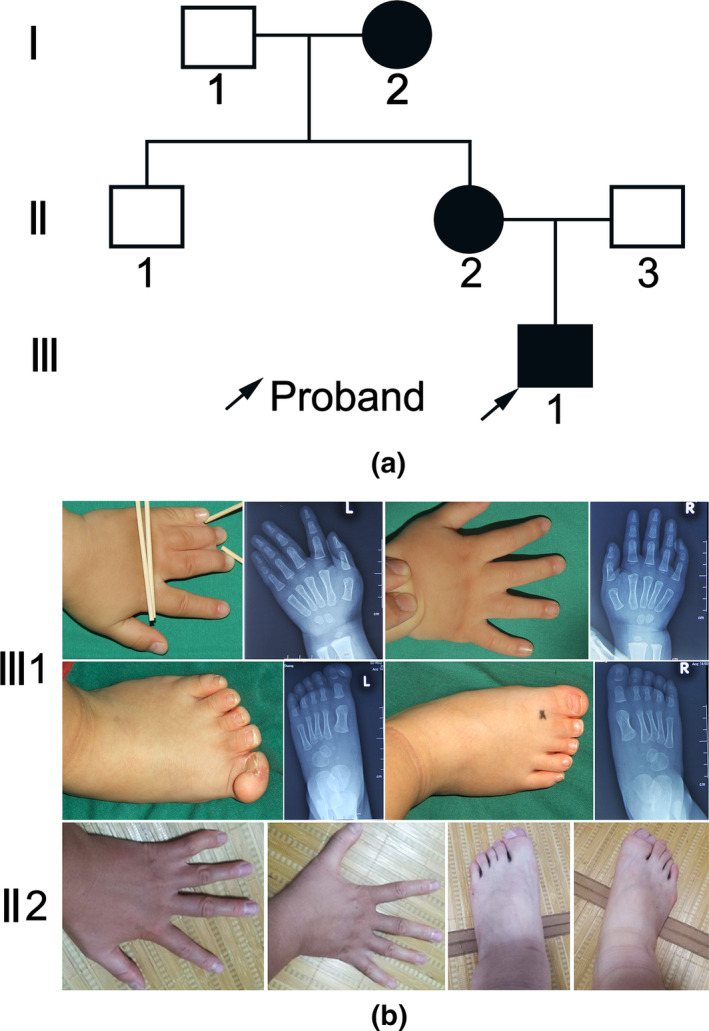
(a) the pedigree of a three‐generation Chinese family with PSD. Arrow represented proband. Circles and squares represented female and male, respectively. Blank and black represented unaffected and affected member, respectively. (b) The clinical characteristics of the proband (III 1) and his mother (II 2)

The pedigree of the Chinese family with PSD was shown in Figure 1a, was close of preaxial polydactyly type IV. In this family, this phenotype affected three successive generations. The proband was a 1.5‐year‐old boy with PSD at both hands and foots since he was born (Figure 1b). A simple and incomplete syndactyly was noted at the middle, ring, and little fingers in his left hand. Ulnar polydactyly of little finger (only finger bud) was also observed. Radial polydactyly of thumb showed only an incomplete division at the distal phalanx, and finger appearance was normal. There was syndactyly at the toes 1, 2, 3, and 4 and polydactyly of big toe in his left foot. Syndactyly at the toes 1, 2, and 3 was also found in his right foot. The second affected member was the mother of proband. A simple and incomplete syndactyly was noted at the ring and little fingers in her both hands. Duplicated hallux and syndactyly between toes 2 and 3 were observed in both feet. The third affected member was the grandma of proband. Her hands had no visible abnormalities and her feet appeared the same phenotype as the second affected member (no pictures were captured). Apart from PSD, no other abnormalities were found. The clinical features of the three affected subjects were presented in Table 2.

**Table 2:** The clinical features observed in the three affected subjects

Individuals	Sex	Age	Features			
			Left hand	Right hand	Left foot	Right foot
Ⅰ−2	F	52	—	—	PreP, SIS	PreP, SIS
Ⅱ−2	F	27	SIS	SIS	PreP, SIS	PreP, SIS
Ⅲ−1	M	1.5	PreP, PostP, SIS,	PreP, SIS	PreP, SIS	SIS

Abbreviations: F, female; M, male; PreP, preaxial polydactyly; PostP, postaxial polydactyly; SIS, simple and incomplete syndactyly; —, feature absent.

### 3.2 Mutation analysis

Whole exome sequencing was performed to detect DNA samples from affected and unaffected family members. The raw image data were obtained and transformed to sequenced reads that was named as raw data. After removing unqualified reads, such as adapter contamination, low‐quality nucleotides, and unrecognizable nucleotide, clean data were obtained and mapped to the reference human genome (UCSC, hg19) to generate BAM files by BWA software. SNPs and InDels were identified by Samtools and GATK software, and were annotated by ANNOVAR. According to priority score and quality score for mutations, 147 SNPs and 15 InDels remained. Considering the important effects of InDels on gene function, we selected 3 InDels with higher quality score, including *GLI3* (NM_000168:c.4659del, NP_000159.3: p.Ser1553del), *ANKUB1* (NM_001144960:c.1385del, NP_001138432.1: p.Pro462del), and *TAS2R3* (NM_016943:c.128_131del, NP_058639.1: p.Leu43del). All of these three mutations were heterozygous, and the allele frequencies were listed in Table 3.

**Table 3:** The alleles frequency of the detected variants

Gene	Position	Alleles	Frequency		
			1000G	ExAC	GnomAD
ANKUB1	Chr3:149485064 (GRCh37.p13)	Del G	—	—	—
TAS2R3	Chr7:141464088–141464091 (GRCh37.p13)	Del TCTG	—	0.00001	0.00001
GLI3	Chr7:42004012 (GRCh37.p13)	Del G	—	—	—

Note

—; no information.

**Figure 2:**
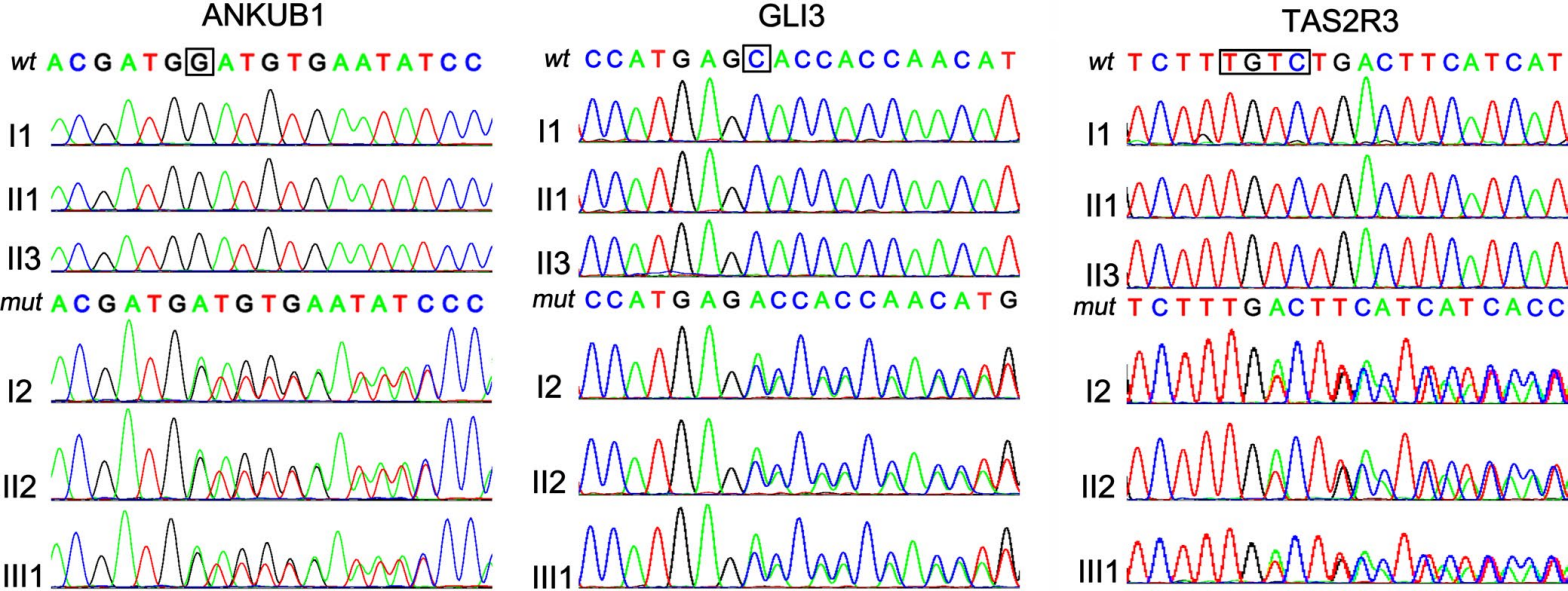
Novel frameshift mutations of *ANKUB1*, *GLI3,* and *TAS2R3*. Sanger sequencing was performed to verify the mutations in *ANKUB1*, *GLI3,* and *TAS2R3* detected by wholeexome sequencing

Direct DNA sequencing showed that mutations in *ANKUB1*, *GLI3,* and *TAS2R3* were observed in all three affected members, but not in all three unaffected members (Figure 2), suggesting that these mutations might be associated with PSD. Other SNPs identified in this study were listed in supplemental file 1.

### 3.3 GLI3 truncation mutation reduced the transcriptional activity of GLI3

**Figure 3:**
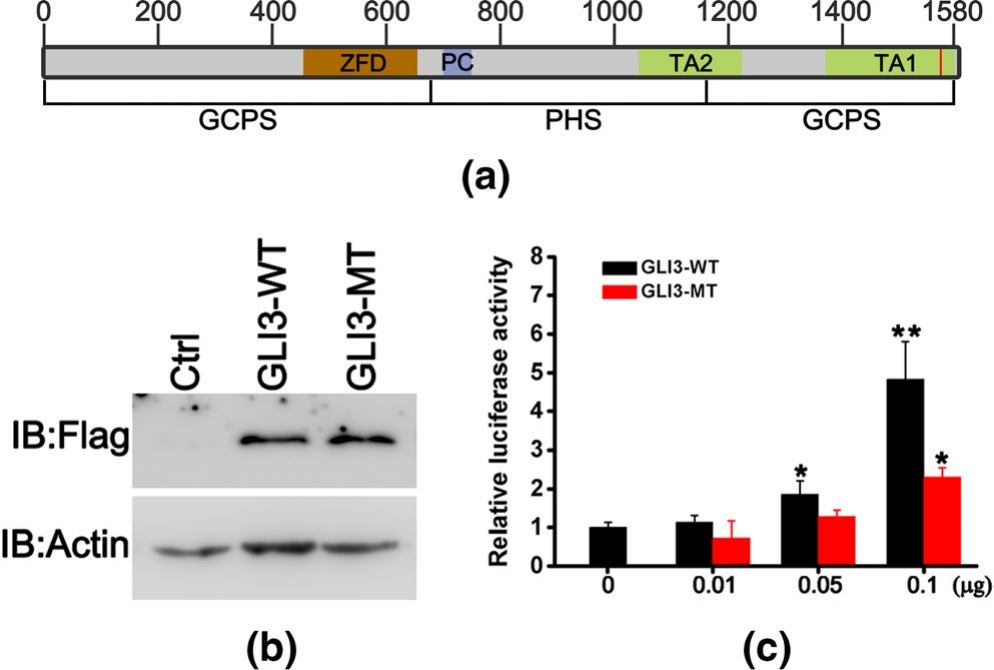
The frameshift mutation of *GLI3* reduced its transcriptional activity. (a) Schematic diagram of GLI3 domains. The red vertical line denoted the mutation site. (b) 293T cells were transfected with flag‐tagged pcDNA3.1‐GLI3‐WT or flag‐tagged pcDNA3.1‐GLI3‐MT. The GLI3 expression was detected using antiflag antibody. (c) 293T cells were cotransfected with pGL3‐PTCH1, and indicated concentration of pcDNA3.1‐GLI3‐WT or pcDNA3.1‐GLI3‐MT using Lipofectamine 3,000. Luciferase activities were determined using the Dual‐Luciferase Assay (Promega) according to the manufacturer's instructions. Data represent means ± *SD* of at least three independent experiments. *p < .05 versus control; **p < .01 versus control

Figure 3a illustrated that GLI3 protein is comprised of three parts, among which the N‐terminal part contains the zinc finger domain (ZFD, AA, 462–645), the middle part contains the protein cleavage site (PC, AA, 703–740), and the C‐terminal part contains two transactivating domains (TA2, AA, 1044–1322 and TA1, AA, 1376–1580). Mutations in N‐terminal part cause GCPS phenotype, mutations in middle part lead to PHS, and mutations in C‐terminal part result in a loss of activator function in a grade manner, inducing GCPS and polydactyly. Considering that GLI3 is a key transcription factor regulating limb development, we explored whether *GLI3* mutation (exon15:c.4659delC, p.S1553fs) affected the transcriptional activity of GLI3. As shown in Figure 3b, no significant difference was observed in molecular weight of GLI3 in 293T cells overexpressing flag‐tagged pcDNA3.1‐GLI3‐WT and flag‐tagged pcDNA3.1‐GLI3‐MT. However, overexpression of GLI3‐MT remarkably reduced the luciferase activity mediated by the promoter of PTCH1 compared to overexpression of GLI3‐WT (Figure 3c). These results suggested that GLI3 mutation (exon15:c.4659delC, p.S1553fs) reduced the transcriptional activity of GLI3.

### 3.4 The location of the novel frameshift mutations of ANKUB1 and TAS2R3

**Figure 4:**
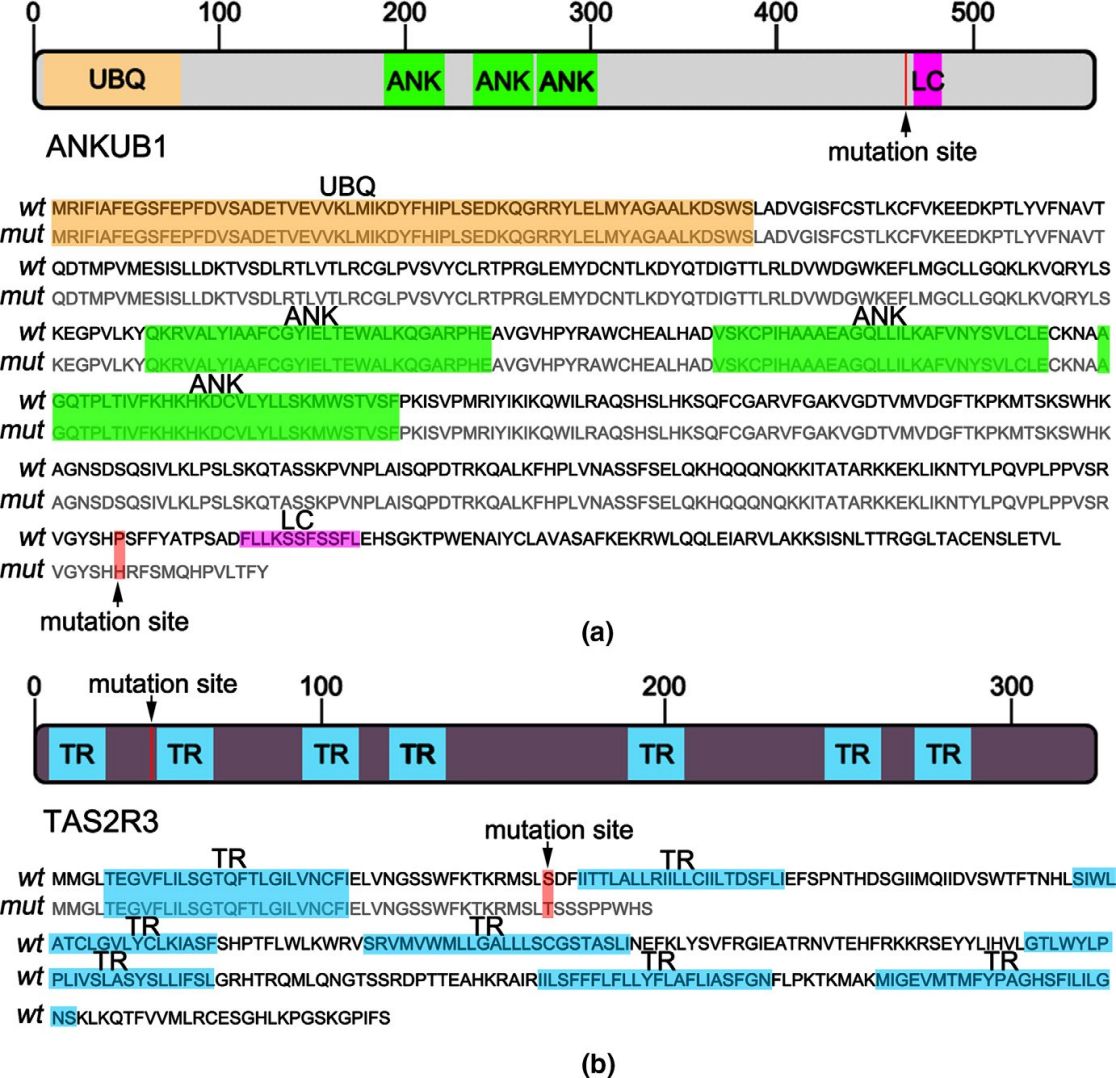
The location of *ANKUB1* and *TAS2R3* mutations. (a) Schematic diagram and protein sequence of ANKUB1 domains and location of *ANKUB1* mutation identified in this study. (b) Schematic diagram and protein sequence of TAS2R3 domains and location of *TAS2R3* mutation identified in this study

We further mapped the putative structural domain according to the protein sequence using SMART analysis (http://smart.embl‐heidelberg.de/). Figure 4a illustrated that ANKUB1 was comprised of three ankyrin repeat domains (ANK), one ubiquitin domain (UBQ), and one low complexity (LC). The mutation of ANKUB1 located in front of the LC domain, which resulted in the deficiency of this domain. It was necessary to validate the role of this ANKUB1 mutation in the function of ANKUB1. Figure 4b showed that TAS2R3 contains multiple transmembrane regions. The frameshift mutation of TAS2R3 located in region prior to the second transmembrane domain, which deleted most amino acid sequence of TAS2R3 (after 43rd amino acid). Therefore, it was likely that this mutation destroyed the protein function.

## 4 DISCUSSION

PSD is most common limb deformity and is regulated by multiple genes (Goodman, 2002). *HOXD13* mutations have widely been implicated in PSD (Brison et al., 2014). Recently, *GLI3* mutations are also identified in human congenital malformation (Al‐Qattan et al., 2017). GLI3 is one of glioma‐associated oncogene family members, and acts as a transcription factor that regulates cell proliferation, death, and differentiation (Li, Zhang, Choi, Litingtung, & Chiang, 2004). Therefore, mutations of *GLI3* gene cause several adverse developmental consequences. *GLI3* mutations have been demonstrated to be closely associated with GCPS, PHS, and isolated polydactyly (Al‐Qattan, 2012; Al‐Qattan et al., 2017; Demurger et al., 2015). The first mutation of *GLI3* has been found in 1991 (Vortkamp, Gessler, & Grzeschik, 1991). Since then, a total of 223 mutations are identified in many kinds of human genetic diseases (Stenson et al., 2017). GLI3 protein can be functionally divided into the N‐terminal part, the middle part, and the C‐terminal part. The N‐terminal part contains the zinc finger domain (ZFD, AA, 462–645), the middle part contains the protein cleavage site (PC, AA, 703–740), and the C‐terminal part contains two transactivating domains (TA2, AA, 1044–1322 and TA1, AA, 1376–1580) (Demurger et al., 2015). Mutations in N‐terminal part mainly lead to GCPS phenotype, mutations in middle part can give rise to PHS, and mutations in C‐terminal part can result in GCPS and polydactyly. In the present study, we found a frameshift mutation of *GLI3* in a pedigree of the Chinese family with PSD (preaxial polydactyly type IV). This frameshift mutation was a novel InDel mutation that had no information in 1000Genomes, ExAC, and GnomAD_exome databases, which caused a change of 28 amino acids in C‐terminal part of GLI3. Luciferase reporter assay demonstrated that this frameshift mutation reduced the transcriptional activity of GLI3. It has been demonstrated that GLI3 possesses a dual function, including a transcriptional activator of SHH signaling pathway by phosphorylated full‐length GLI3 and a repressor by C‐terminally truncated CLI3. Recently, several novel mutations of GLI3 have been identified in the patients with PD, including mutation c. 1622C > T (Zou et al., 2019), c.2148delA (Zhao, Xu, Liu, & Li, 2019), c.3437_3453delTCGAGCAGCCCTGCCCC, and c.3997C > T (Chen et al., 2019), c.1180C > TT (Ni et al., 2019). Therefore, our results suggested that this frameshift mutation of *GLI3* might be a main reason for preaxial PD type IV phenotype in this Chinese family.

We also identified other gene mutations, such as *ANKUB1* and *TAS2R3*. The frameshift mutations of *ANKUB1* and *TAS2R3* have never been identified to be associated with SPD. The full name of ANKUB1 is ankyrin repeat and ubiquitin domain containing 1, also known as C3orf16. As the name implies, ANKUB1 contained ankyrin repeat domain and ubiquitin domain. We mapped the putative structural domain according to the protein sequence using SMART analysis (http://smart.embl‐heidelberg.de/). As shown in Figure 4a, ANKUB1 was comprised of three ankyrin repeat domains (ANK), one ubiquitin domain (UBQ), and one low complexity (LC). Ankyrin repeat domain is a 33‐residue motif that is frequently found in vertebrate proteins, mediating the protein–protein interaction. Ubiquitin contains 76 amino acid residues that are extremely conserved in all eukaryotic cells. Ubiquitin affects the function of other proteins by ubiquitinylation. In this study, the frameshift at codon 1,385 (NM_001144960, exon5:c.1385delG, p.P462fs) truncated C‐terminally the ANKUB1 protein, but did not affect ankyrin repeat domain and ubiquintin domain. It is necessary to determine whether this frameshift mutation altered the function of ANKUB1, and affected osteogenesis and osteoclast differentiation. TAS2R3 is a member of type 2 taste receptors (TAS2Rs) that belong to a class of G protein‐coupled receptors (Choi et al., 2018). TAS2R3 contains multiple transmembrane regions (Figure 4b), mediating signal transduction on the cellular membrane. Previous studies mainly focused on the function of TAS2Rs in bitterness sensing (Choi et al., 2018). Recently, genetic variation in *TAS2R3* has been associated with the risk of papillary thyroid carcinoma and regulates thyroid function (Choi et al., 2018). In addition, the SNP of *TAS2R3* has been closely correlated to male infertility (Gentiluomo et al., 2017). In the present study, a frameshift mutation of *TAS2R3* (NM_016943, exon1:c.128_131delTGTC, p.L43fs) was found to be associated with PSD. This mutation altered most amino acid sequence of TAS2R3 (after 43rd amino acid), which probably destroyed the protein function. It would be interesting to determine the role of TAS2R3 in limb development.

## 5 CONCLUSIONS

In summary, we evidenced that a novel frameshift mutation in GLI3 segregates with the malformation in the Chinese family, but novel rare variants in ANKUB1 and TAS2R3 also segregate in this family. Further investigation demonstrated that the frameshift mutation of GLI3 reduced the transcriptional activity of GLI3. According to the results from SMART analysis, the frameshift mutation of TAS2R3 altered most protein sequence, resulting in a damage in protein function. Although the frameshift mutation of ANKUB1 did not affected ankyrin repeat domain and ubiquitin domain, it probably affected the interaction between ANKUB1 and other proteins, and altered the ubiquitinylation. Our results suggested that the frame‐shift mutations of GLI3 reduced its transcriptional activity, contributing to malformation progression. Since the pedigree is small, the causative nature of variants in ANKUB1 and TAS2R3 remains unclear.

## CONFLICT OF INTEREST

All authors have declared that no competing interests exist.

## Supporting information

Supplementary Material

## Data Availability

All data used or analyzed during this study are included in this published article.
